# Co-Expression of Ezrin-CLIC5-Podocalyxin Is Associated with Migration and Invasiveness in Hepatocellular Carcinoma

**DOI:** 10.1371/journal.pone.0131605

**Published:** 2015-07-02

**Authors:** Teresita N. J. Flores-Téllez, Tania V. Lopez, Verónica Rocío Vásquez Garzón, Saúl Villa-Treviño

**Affiliations:** 1 Departamento de Biología Celular, Centro de Investigación y de Estudios Avanzados del Instituto Politécnico Nacional (CINVESTAV-IPN), Av. IPN No. 2508 Col. San Pedro Zacatenco, México 14, CP 07360, México, Distrito Federal; 2 Instituto Nacional De Medicina Genómica (INMEGEN), Periférico Sur 4809, Arenal Tepepan, Tlalpan, 14610 Ciudad de México, Distrito Federal; 3 Facultad de Medicina y Cirugía, Universidad Benito Juárez de Oaxaca. Av Universidad S/N, Col. 5 Señores. C.P. 68120, México, Oaxaca; University of Navarra School of Medicine and Center for Applied Medical Research (CIMA), SPAIN

## Abstract

**Background and Aim:**

Prognostic markers are important for predicting the progression and staging of hepatocellular carcinoma (HCC). Ezrin (EZR) and Podocalyxin (PODXL) are proteins associated with invasion, migration and poor prognosis in various types of cancer. Recently, it has been observed that chloride intracellular channel 5 (CLIC5) forms a complex with EZR and PODXL and that it is required for podocyte structure and function. In this study, we evaluated the overexpression of EZR, PODXL and CLIC5 in HCC.

**Methods:**

The modified resistant hepatocyte model (MRHR), human biopsies and HCC cell lines (HepG2, Huh7 and SNU387) were used in this study. Gene and protein expression levels were evaluated in the MRHR by qRT-PCR, Western blot and immunohistochemistry analyses, and protein expression in the human biopsies was evaluated by immunohistochemistry. Protein expression in the HCC cell lines was evaluated by immunofluorescence and Western blot, also the migration and invasive abilities of Huh7 cells were evaluated using shRNA-mediated inhibition.

**Results:**

Our results indicated that these genes and proteins were overexpressed in HCC. Moreover, when the expression of CLIC5 and PODXL was inhibited in Huh7 cells, we observed decreased migration and invasion.

**Conclusion:**

This study suggested that EZR, CLIC5 and PODXL could be biological markers to predict the prognosis of HCC and that these proteins participate in migration and invasion processes.

## Introduction

Invasion is a critical step in the development of metastasis, which is characterized by changes in the expression of proteins related to the cytoskeleton, migration, and invasion [1]. Several cell membrane and cytoskeleton-related proteins are overexpressed in many types of cancers, including Ezrin (EZR) [2] and Podocalyxin (PODXL) [3], which are associated with morphogenesis and migration because they function as scaffold proteins between the cell membrane and actin cytoskeleton [4, 5]. EZR and PODXL are also overexpressed in various tumors [6–13], and their co-expression enhances the metastatic potential of cell lines and tumors [14, 15]. Chloride intracellular channel 5 (CLIC5) is an actin-associated protein involved in cell projections of placental microvilli [16], inner ear hair cells [17] and podocytes [18]. Recently, it has been observed that CLIC5 forms a complex with EZR and PODXL that is required for podocyte structure and function [19].

Hepatocellular cancer (HCC) has high mortality because patients are asymptomatic until the cancer is so advanced that the tumor cannot be treated with radical hepatectomy and traditional therapy (chemotherapy and radiotherapy). Therefore, there is an urgent need to identify new and effective molecular markers for diagnosis and prognosis prediction and to find new molecular therapeutic targets for HCC patients [20]. Although CLIC5 has not been reported to be involved in hepatocellular carcinogenesis, it has been proposed as a metastatic risk marker due to its overexpression in breast tumors and rectal tumors after radiotherapy [21, 22]. Members of the CLIC family (CLIC1 and CLIC4) are associated with proliferation, angiogenesis, drug resistance, invasion, metastasis and poor prognosis [23–25]. In this study, we examined the expression of EZR, CLIC5 and PODXL at the mRNA and protein levels in a modified hepatocyte resistant model (MHRM) and in cases of human hepatocellular carcinoma (HCC). Additionally, we studied for the first time the roles of CLIC5 and PODXL in HCC cell line migration and invasion.

## Materials and Methods

### Antibodies

Anti-CLIC5, rabbit polyclonal antibody was purchased from Abcam (Cambridge, USA), whereas-PODXL,-EZR and-GADPH rabbit polyclonal antibodies were purchased from Millipore (California, USA) and Cell Signaling Technology (Danvers, USA), respectively. The anti-GSTP goat polyclonal antibody was obtained from Santa Cruz (Texas, USA); HRP-coupled rabbit and goat secondary antibodies were from Invitrogen (California, USA) and ZYMED (California, USA), respectively. The biotinylated secondary antibody was obtained from Dako and the FITC-conjugated secondary antibody from Jackson Immunoresearch Laboratories Inc. (Pennsylvania, USA).

### Cell lines and culture

HepG2 (American Type Culture Collection, ATCC), Huh7 (donated by Dr. Julio Isael Pérez Carreón) and C2C12 (ATCC) cell lines were maintained in Dulbecco’s modified Eagle medium (DMEM; Invitrogen) supplemented with 10% fetal bovine serum (FBS) (Gibco) and penicillin/streptomycin (Invitrogen). SNU387 (ATCC) and HEK293 (donated by Dr. Zentella Dehesa) cell lines were maintained in RPMI 1640 (Invitro) containing 10% FBS. C2C12 (ATCC) cells were used as a positive control for CLIC5 expression; HEK293 cells were used as a positive control for EZR and PODXL expression. All cells were incubated at 37°C in a humidified atmosphere of 95% air/5% CO_2_.

### Clinical samples

Paraffin-embedded samples from HCC cases (4 females and 5 males; a mean age of 54.4 years, range of 17–70 years) were donated by Rebeca García Román (Universidad Veracruzana) and processed and reviewed by expert pathologists. Written informed consent was obtained from all of the patients before under going, in which patients agreed to use their medical record data and tissue samples before research. This work was performed in accordance with the following entities: Declaration of Helsinki (2000) of the World Medical Association; the Mexican General Health Law (2011); the Regulation of the General Law on Health Research for Health (1987) and the Health Law of the State of Veracruz-Llave (2008). This study was reviewed and approved by the Scientific and Ethical Hospital Committee (Centro de Especialidades Médicas del Estado de Veracruz, CEMEV) (permission number: 005/2011). Our analysis parameters for images included the number of objects defined as cells in the image, staining intensity (high, medium, low and negative) and fraction (%) of positive cells in the image area.

### Animals and treatments

All experiments were performed in accordance and approved by the Institutional Animal Care and Use Committee named “Comité Interno para el Cuidado y Uso de Animales de Laboratorio (CICUAL)” under protocol No. 0001–02. Male Fischer 344 rats were acquired from the Unit for Production of Experimental Laboratory Animals (UPEAL Cinvestav, Mexico City, Mexico) and were given free access to food (PMI Feeds Inc., Laboratory Diet) and water, and they were maintained in a holding room under controlled conditions with a 12 h light/dark cycle, 50% relative humidity, and a temperature of 21°C. Male Fischer 344 rats weighing 180–200 g were subjected to a previously reported carcinogen treatment: briefly, rats were initiated with an intraperitoneal dose of DEN (200 mg/kg; Sigma-Aldrich), the administration of 2-AFF (25 mg/kg) was in three consecutive days, beginning on day 7 after initiation. On day 10, rats were subjected to parcial hepatectomy (HP) [26]. Animals of each group (n = 4); experimental and control groups, were sacrificed at 9, 12 and 18 months, and their livers were excised and washed with phosphate-buffered saline (PBS). A section was cryopreserved in liquid nitrogen for RNA and protein extraction, and additional sections were fixed in 4% paraformaldehyde in PBS and processed for routine histological examination. Complete Arrive guidelines Checklist in included in S1 Table [27].

### Histological analyses

For detection of nodular, tumor and non-tumor areas, the frozen rat livers were cut into 20-μm sections, and γ-glutamyl transferase (GGT) activity was evaluated as described [28].

### RNA isolation and qRT-PCR

Total RNA was isolated from nodular, tumor and non-tumor areas of liver tissue using TriPure Isolation Reagent (Roche) according to the manufacturer’s protocol. cDNA synthesis was performed with 1 μg of purified RNA and 1 μL of Superscript II Reverse Transcriptase (Invitrogen). The expression levels of the target genes EZR, PODXL and CLIC5 were measured by qRT-PCR amplification performed using the TaqMan Gene Expression Assay in a Step One Real-Time PCR System (Applied Biosystems, Foster City, CA, USA). The 18s rRNA (Rn18s) gene was used as an internal control. The PCR conditions were 50°C for 2 min, 95°C for 10 min and 40 cycles at 95°C for 1 sec followed by 60°C 1 min. The relative expression levels were calculated using the 2^-ΔΔCt^ method.

### Immunoprecipitation and Western blot analysis

For protein isolation, samples from nodular, tumor and non-tumor areas were lysed in buffer containing Complete Protease Inhibitor Cocktail (Roche Applied Science, Manheim, Germany) and phosphatase inhibitors (Roche Applied Science, Penzberg, Germany). Protein extract was obtained from cells using lysis buffer containing protease and phosphatase inhibitors. For immunoprecipitation assays, cells were lysed using the Pierce Co-IP kit (Thermo Scientific, Rockford, USA) according to the manufacturer's instructions. Proteins were resolved by 10% SDS-PAGE, transferred to PVDF membranes (Millipore, USA), incubated with the appropriate primary and secondary antibodies and detected using the ImmunoCruz Western Blotting Luminol Reagent (Santa Cruz, USA). Densitometry analysis was performed with ImageJ software.

### Immunohistochemistry

For immunostaining, liver sections (3 μm) were incubated for 20 min with 10 mM sodium citrate buffer (pH 6.0) and then with 1% H_2_O_2_ for 20 min to quench endogenous peroxidase activity. Nonspecific protein binding was blocked with CAS-block solution (Invitrogen, Paisley, UK). The sections were incubated overnight with the appropriate primary and secondary antibodies. The signals were detected using the DAB kit (Invitrogen, California, USA).

### Immunofluorescence microscopy

Briefly, cells were fixed with 4% paraformaldehyde in PBS for 15 min; nonspecific binding was blocked by 5% serum and 0.3% Triton X-100 in PBS for 1 h. The cells were incubated overnight at 4°C with primary antibodies followed by incubation with a FITC-conjugated secondary antibody and staining with Alexa Flour 555 Phalloidin (8953, Cell Signaling, Danvers, USA) and Hoechst solution (Sigma, USA). The cells were mounted on coverslips using Vectashield mounting medium (Vector Laboratories Inc., California, USA). Images were acquired with a Leica TCS SP8 confocal microscope.

### shRNA inhibition of CLIC5 and PODXL expression

To inhibit PODXL and CLIC5 expression, pools of concentrated transduction-ready viral particles containing 3 target-specific shRNAs (19–25 nt, plus hairpin) designed to knock down gene expression were used to transfect cells following the manufacturer’s instructions (Santa Cruz, USA). Scrambled shRNA served as a negative control. Proteins were examined by Western blot analysis.

### Wound healing assay

Control and PODXL- and CLIC5-knockdown Huh7 cells were grown in 35-mm culture dishes (Santa Cruz, USA) until confluence. The cells were wounded using a sterile pipette tip and then washed twice with PBS. Images were taken at 24, 48 and 72 h. Image analysis was performed with ImageJ software.

### MTT assay

Cells (1x10^4^) were grown in a 96-well plate, and proliferation was measured by the methylthiazolyldiphenyl-tetrazolium (MTT) assay (Sigma, Missouri, USA) after 48 h.

### Migration and invasion assays

Using Millipore kits (Massachusetts, USA), control and PODXL- and CLIC5-knockdown Huh7 cells (1x10^5^) were seeded in serum-free medium in the upper chamber and incubated for 24 and 36 h for migration and invasion assays, respectively. The migrated and invaded cells were quantified according to the manufacturer's instructions.

### Statistical analysis

The results are presented as the mean ± standard error of the mean (SEM). Differences between control and treatment groups (rats; each group n = 4; cells; each group n = 4) were analyzed using ANOVA and the Tukey–Kramer post-test. Ninety-five percent confidence intervals (CIs) and p values were calculated. For each test, p<0.05 was considered significant.

## Results

### EZR, CLIC5 and PODXL are highly expressed in rat liver tumors

**Fig 1:**
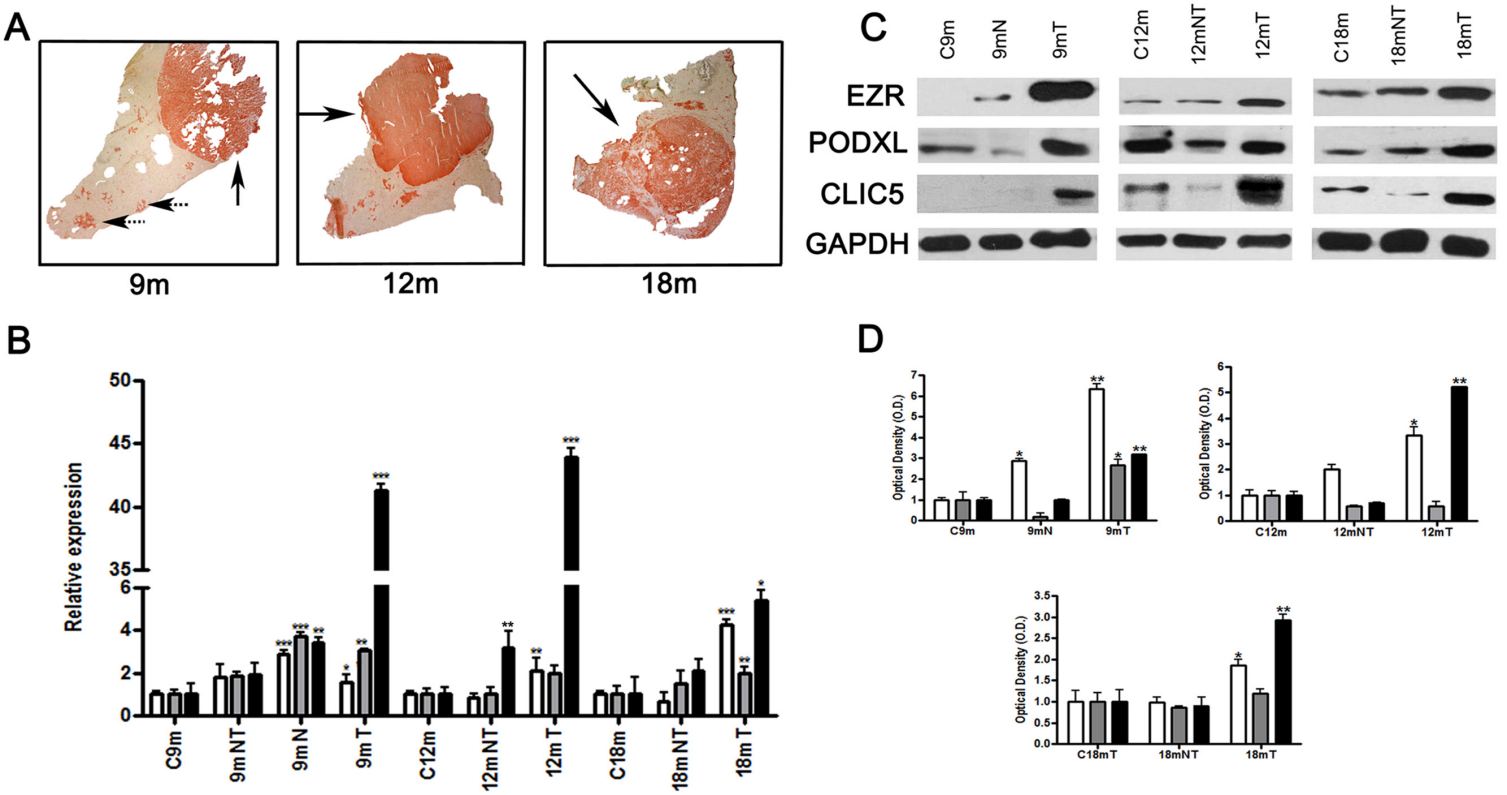
Expression of EZR, PODXL and CLIC5 in rat livers after carcinogenic treatment. Male rats used in the MRHM were sacrificed at 9 (9 m), 12 (12 m) and 18 (18 m) months. A) Tumor and preneoplastic lesions were characterized by GGT activity (spotted and solid arrows, respectively). B) Analysis of relative expression of genes by qRT-PCR in rat livers. C) Protein expression in fractions isolated from rat livers was assessed by Western blot analysis, representative image. D) Graphic representation of triplicates of western blot. GADPH was included as a loading control. C9 m, C12 m and C18 m represent controls of each time point; 9 mT, 12 mT and 18 mT represent tumor regions; 9 mN represents nodular areas (preneoplastic lesions); and 9 mNT, 12 mNT and 18 mNT represent non-tumor areas of the liver (n = 4 rats per group). □EZR, ∎PODXL and ■CLIC5. Error bars indicate the standard error of the mean (SEM). *p≤0.05, **p≤0.03 and ***p≤0.002 vs. their respectively untreated control values (ANOVA and Tukey–Kramer test).

As we reported previously, the carcinogen treatment in MHRM produced nodules and tumors in rats after 9, 12 and 18 months; the experimental group developed nodules and tumors (4/4 rats) and the control group did not present tumors (0/4 rats) [26] (S1 Fig). The nodular (dotted arrow) and tumor areas (solid arrow) in the liver exhibited GGT activity (GGT+), whereas normal hepatocytes did not (GGT-) (Fig 1A). To analyze the liver expression of EZR, CLIC5 and PODXL, we isolated GGT+ or GGT- areas for mRNA extraction and then subjected the mRNA to qRT-PCR. Overexpression of EZR, CLIC5 and PODXL genes was observed in nodular (N) and tumor (T) areas but not in non-tumor (NT) areas and controls (C). For EZR and PODXL, the highest relative expression was in tumor areas at 18 months, with values of 4.51 (p<0.002) for EZR and 3.72 (p<0.03) for PODXL. CLIC5 showed high relative expression at 9 months, with a value of 41.27 (p<0.002), and at 12 months, with a value of 43.97 (p<0.002), but was only 5.43 (p<0.05) at 18 months (Fig 1B), although such a large CLIC5 overexpression is no observed in 18mT compared to 9mT and 12mT; it remains higher compared to 18m control. The protein expression levels of EZR, CLIC5 and PODXL were higher in nodular and tumor areas compared to non-tumor areas or controls (p<0.05), which correlated with the qRT-PCR results (Fig 1C and 1D).

### EZR, CLIC5 and PODXL co-localize in tumor regions

**Fig 2:**
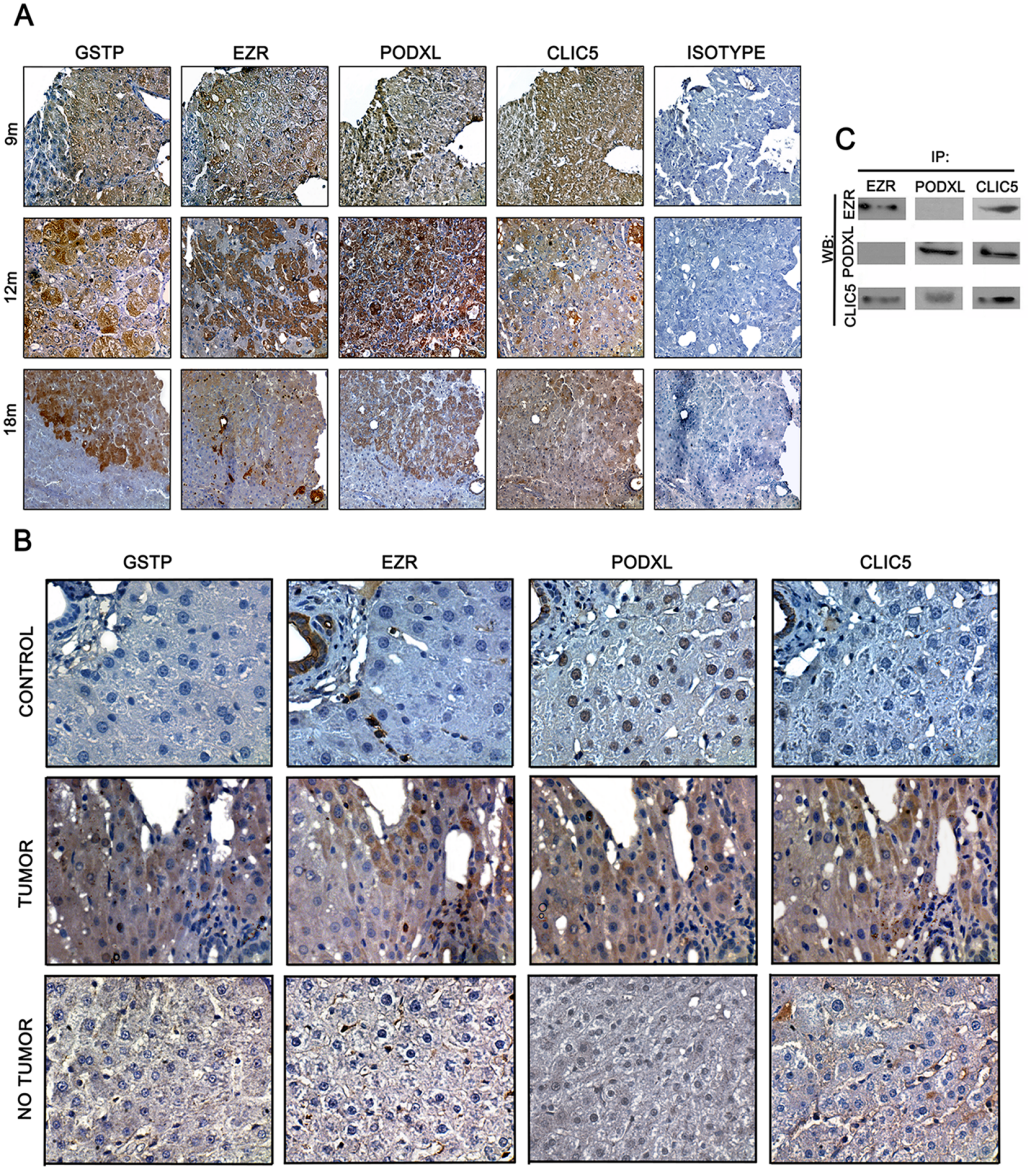
Co-localization of EZR, CLIC5 and PODXL in tumors of rat livers after carcinogenic treatment. Male rats used in the MRHM were sacrificed at 9 (9 m), 12 (12 m) and 18 (18 m) months. A) EZR, CLIC5 and PODXL expression was analyzed by immunohistochemistry in 3 μm liver serial sections. GSTP detection was used to identify tumor areas (40x magnification). B) Differential expression of EZR, PODXL and CLIC5 in tumor and non-tumor areas of rat livers after carcinogenic treatment. Protein expression was also analyzed in non-treated rats (control) (60x magnification). C) Interaction among CLIC5, EZR and PODXL at 18 m as analyzed by co-immunoprecipitation.

We analyzed EZR, CLIC5 and PODXL expression by immunohistochemistry (Fig 2). Using GSTP as a marker for neoplastic lesions and tumors areas [29], EZR, CLIC5 and PODXL expression was co-localized within the tumor region of serial samples of rat liver tumors after 9, 12 and 18 months of treatment (Fig 2A). In tumor regions, EZR expression was observed in the cytoplasm of hepatocytes, but EZR was not expressed in non-tumor regions; cells in the sinusoidal space did express EZR. Similarly, EZR expression was found in cells of the sinusoidal space in livers from non-treated rats. CLIC5 was detected in the cytoplasm of tumor region hepatocytes but was not expressed in controls and was only weakly expressed in non-tumor regions of livers from treated rats. PODXL was detected in the cytoplasm of tumor-region hepatocytes from treated rats, whereas PODXL expression was clearly nuclear in control livers. Interestingly, in non-tumor regions of livers from treated rats, PODXL was weakly observed in hepatocyte nuclei (Fig 2B). Weak signals of EZR and CLIC5 were detected in the nodular regions of some livers from treated rats, though a clear PODXL signal was always observed in these regions (S2 Fig). All antibodies were also evaluated in tissue controls (S3 Fig).

### CLIC5 interacts with PODXL and EZR

Because we observed that the three proteins co-localized in tumor regions, the interaction of these proteins was evaluated in 18-month tumors by immunoprecipitation and Western blot analysis. CLIC5 was detected when proteins were immunoprecipitated using either anti-EZR or anti-PODXL antibodies. However, EZR and PODXL were detected only when the complex was immunoprecipitated using an anti-CLIC5 antibody (Fig 2C).

### EZR, CLIC5 and PODXL are expressed in human HCC

**Fig 3:**
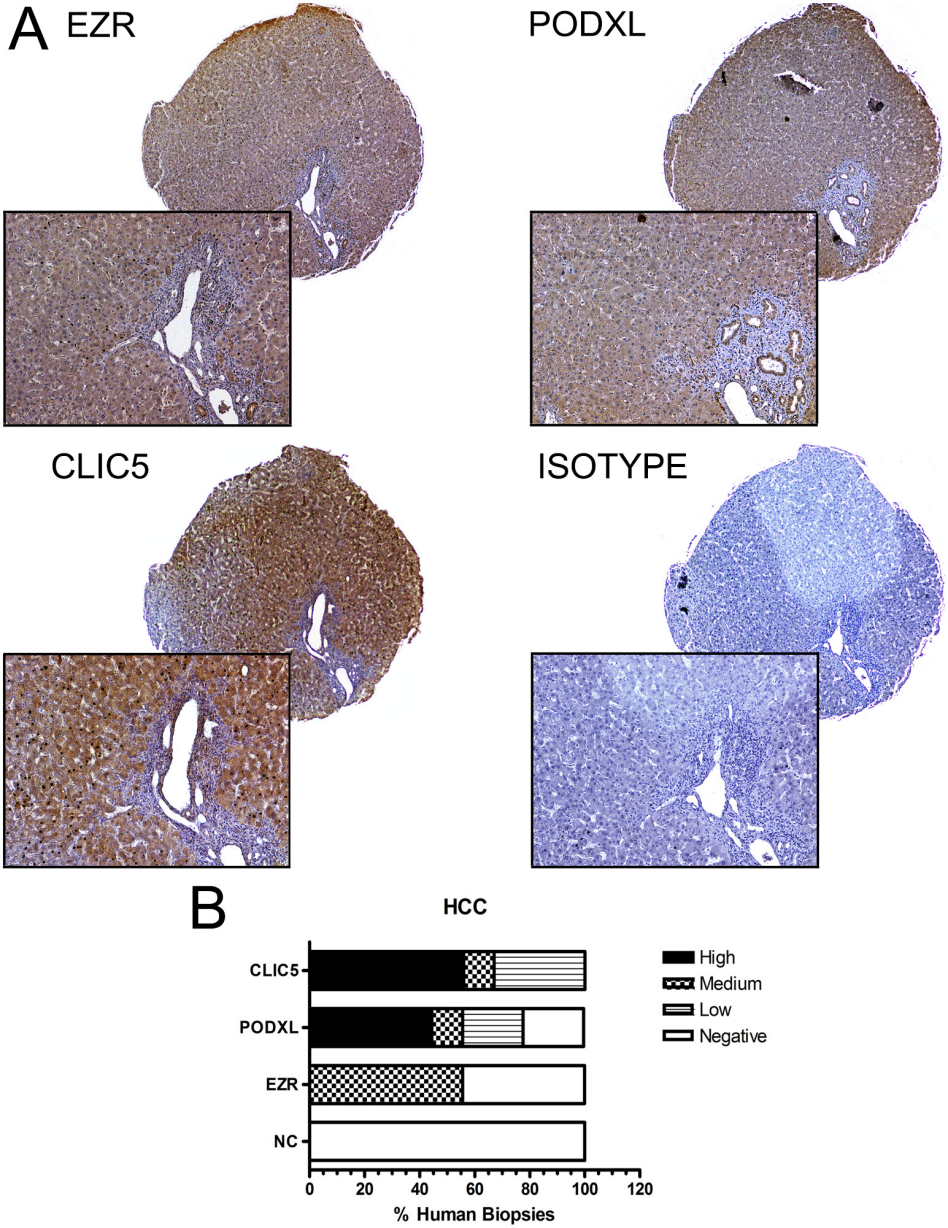
Differential expression of EZR, CLIC5 and PODXL in human biopsies. A) EZR, CLIC5 and PODXL proteins were detected by immunohistochemistry in 3 μm sections from hepatocellular carcinoma (HCC) tissue and negative control (NC) samples (4x and 20x magnification, respectively). B) The graph represents composite information to facilitate the interpretation of all the samples that were positive for each protein. Each biopsy was classified as high intensity, medium intensity, low intensity or negative signal. Representative images of EZR, PODXL and CLIC5 are shown.

Using immunohistochemistry and normal cervix as a negative control (NC), we found that EZR was positive in 5 of 9 HCC biopsies, with 55% exhibiting medium intensity and 44.44% negative. CLIC5 was positive in 9 of 9 HCC biopsies, with 56% staining with high intensity, 11% with medium intensity and 33% low with intensity. Finally, PODXL was positive in 7 of 9 cases, with 44.44% exhibiting high intensity, 11.11% medium intensity, 22% low intensity and 22% negative. CLIC5 was more prominently expressed in HCC than EZR and PODXL (Fig 3A and 3B). Additional details about each human biopsy are noted in S2 Table.

### EZR, CLIC5, and PODXL are expressed in human HCC cell lines

**Fig 4:**
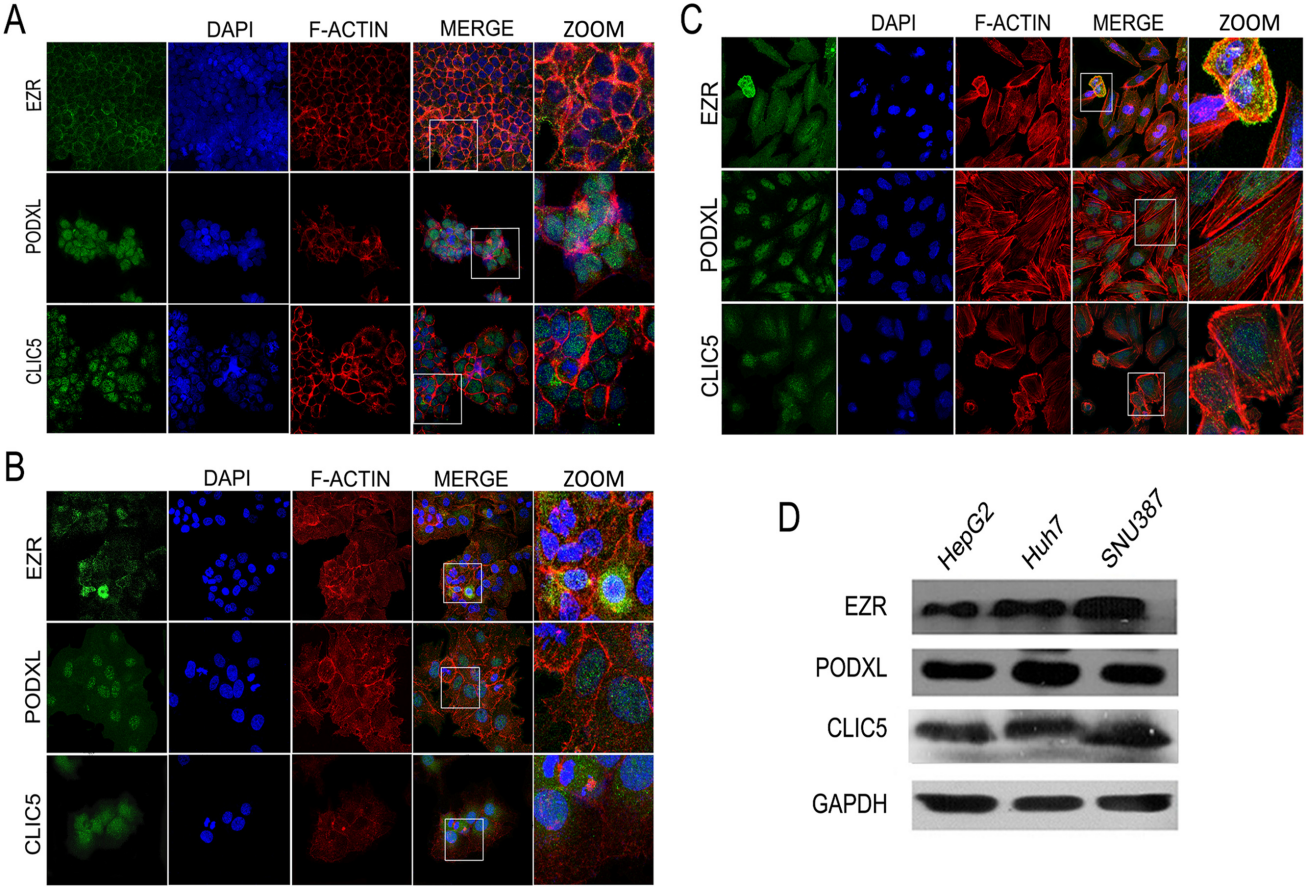
Expression and localization of EZR, PODXL and CLIC5 in HCC cell lines. Localization of EZR, PODXL and CLIC5 in A) HepG2, B) Huh7 and C) SNU387 cell lines. Positive signals for each protein are shown in green. F-actin is shown in red, and nuclei are shown in blue. Magnified images are shown (ZOOM). D) Total protein expression of EZR, PODXL and CLIC5 in the three cell lines was evaluated by Western blot analysis. Each experiment was repeated at least four times.

We evaluated the expression and subcellular localization of the proteins in HCC cell lines using immunofluorescence (Fig 4). In all three cell lines, EZR was expressed in the cytoplasm and around the cell edges and also co-localized with actin; CLIC5 was localized in the cytoplasm. PODXL was located in the nucleus in HepG2 cells but was located in both the nucleus and cytoplasm in SNU387 and Huh7 cells (Fig 4A–4D; S4A–S4C Fig).

### PODXL and CLIC5 expression is associated with invasiveness and cell migration

**Fig 5:**
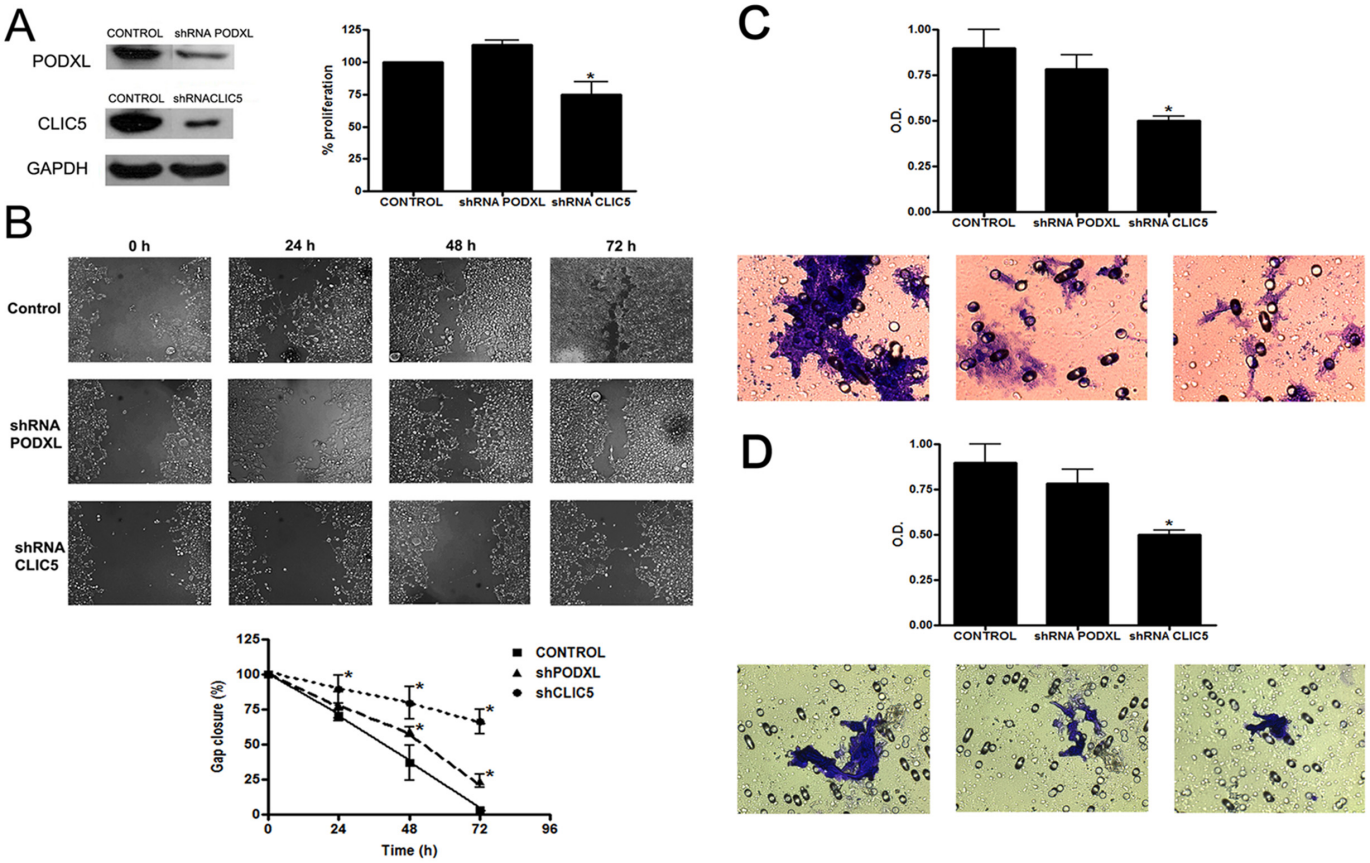
Downregulation of PODXL and CLIC5 affects migration and invasion in Huh7 cell lines. A) (Left) Expression of PODXL and CLIC5 was examined by Western blot analysis in transduced Huh7 cells. GADPH was included as a loading control. (Right) Proliferation of control and transfected Huh7 cells was analyzed using an MTT assay. B) (Up) Representative images of the wound assay in Huh7, shRNAPODXL and shRNACLIC5 cells at 0, 24, 48 and 72 h (20x magnification) (Down) Quantification of gap closure. The graph represents the wound width as the mean in % of the closure of original wound at 0, 24, 48 and 72h. Similar results were obtained in quadruplicate plates. C) The migration capacities of Huh7, shRNAPODXL and shRNACLIC5 cells were measured using the cell migration assay. The migrated cells were fixed, stained with crystal violet, and measured using colorimetry. D) The invasion capacities of Huh7, shRNAPODXL and shRNACLIC5 cells were measured using the ECMatrix Cell Invasion assay. The invasive cells were fixed, stained with crystal violet, and measured using colorimetry. Error bars indicate the standard error of the mean (SEM). *p≤0.05 vs. untreated control values (Tukey–Kramer test). Each experiment was repeated at least four times.

After confirming downregulation by Western blot analysis, we then evaluated the effect of silencing CLIC5 and PODXL in Huh7 cells (Fig 5). CLIC5 downregulation significantly reduced proliferation by 20% compared with the control (p<0.05) (Fig 5A). In the wound assay, CLIC5 and PODXL downregulation significantly (p<0.05) avoided the gap closure of the cells, with CLIC5 downregulation having a more pronounced effect (Fig 5B). The migration assay was performed using transwell migration chambers. Downregulation of CLIC5 and PODXL expression reduced the cell migration potential, with only CLIC5 downregulation having a statistically significant effect (p = 0.01) (Fig 5C). The invasion capacity of Huh7 cells was significantly inhibited by silencing CLIC5 (p = 0.05) (Fig 5D).

## Discussion

Although the clinical staging system for HCC is implemented, there is a need to refine and complement this system with outcome predictions. New biomarkers will improve the prognostic assessment of HCC patients, and we hypothesized that EZR, CLIC5 and PODXL can serve as HCC biomarkers because they participate in HCC cell migration and invasion.

EZR, CLIC5 and PODXL are present in the cytoplasm of hepatocytes in liver tumors from rats and human HCC biopsies. Our study is the first to report overexpression of CLIC5 and PODXL in tumors in addition to their co-localization with EZR. Previous studies have reported EZR or PODXL overexpression in different tumors [6–12], indicating their participation in tumor progression. EZR and PODXL were immunoprecipitated using an anti-CLIC5 antibody, which suggests that CLIC5 could be a scaffold protein between EZR and PODXL in hepatic rat tumors. The function of CLIC5 is proposed to be similar to that of EBP50, which is responsible for the interaction between EZR and PODXL in renal carcinoma [14]. In breast cancer cells, the co-expression and interaction of EZR and PODXL increases the metastatic potential of the cells.

Interestingly, in control livers, EZR, CLIC5 and PODXL did not co-localize because EZR and CLIC5 are not expressed in normal hepatocytes. EZR was expressed in the cytoplasm of sinusoidal space cells, such as Kupffer cells, lymphocytes and bile ducts cells, but not in hepatocytes, consistent with previous reports [30, 31], [32, 33]. In untreated rats, PODXL is expressed in the nuclei of hepatocytes and in the nuclei of A-431, U-251 and U2OS cells, but the report describing these results provided no explanation for this phenomenon [34].

Currently, there are no reports of CLIC5 expression in the liver under pathological conditions, thus making ours the first observation of this effect. Using GSTP staining to identify preneoplastic lesions and by serial immunohistological analysis, we localized the expression of EZR and CLIC5 in preneoplastic lesions in only certain hepatocytes. We hypothesize that such hepatocytes may be those that eventually form the tumor. Our data demonstrated that EZR, CLIC5 and PODXL co-localization in the liver only occurs under the studied pathological conditions, suggesting that these proteins are potential biomarkers of liver cancer. We also observed overexpression of these proteins and a tendency of EZR, CLIC5 and PODXL co-localization in human HCC biopsies.

It has been reported that the cytoplasmic expression of EZR correlates with the metastatic potential of various cell lines [35, 36]. Our study using three HCC-derived cell lines is the first report to evaluate CLIC5 and PODXL expression in these cell lines, and our results suggest that CLIC5 is expressed in the cytoplasm and nuclei in all three cell types. No significant changes were detected in the level of PODXL protein expression among the cell lines. However, nuclear PODXL expression was found in HepG2 cells, whereas both nuclear and cytoplasmic PODXL expression was found in Huh7 and SNU387 cells. Taken together, these findings suggest that the EZR, CLIC5 and PODXL proteins are overexpressed during cell transformation and may undergo changes in subcellular localization during this process.

CLIC5 was initially described as a chloride ion channel, but it has little or no channel activity in vivo [37]. However, CLIC5 overexpression does induce 3T3-L1 cell proliferation and inhibit the expression of proteins associated with the differentiation of adipocytes [38]. In our study, the downregulation of CLIC5 expression reduced Huh7 cell proliferation. In contrast, it has been shown that CLIC5 overexpression in C2C12 cells is associated with the inhibition of proliferation and promotion of differentiation [39].

The expression of EZR and PODXL proteins is associated with increased metastatic potential [15, 40, 41]. We showed that downregulating CLIC5 decreased the migration and invasiveness of Huh7 cells, which may also be associated with both EZR and PODXL. Therefore, we propose that CLIC5 functions as a scaffold protein between these two proteins. Previous studies have shown that EZR inhibition in HCC cells decreases their migratory and invasive potential [35, 42]. In renal cancer, the interaction between EZR and PODXL occurs through EBP50, which serves as a scaffolding protein. Interestingly, the metastatic potential of renal carcinoma cells is significantly reduced when the PDZ1 and/or PDZ2 domains of EBP50 are absent [23]. In liver cancer, CLIC5 might play a similar role because the inhibition of CLIC5 decreases the migration and invasion of Huh7 cells.

Although we observed co-localization of EZR, CLIC5 and PODXL in HCC samples, this expression pattern in tumor cells was only found in the late stage of HCC. Further experiments are needed to understand the biological importance of the interaction of these proteins in cancer. Finally, it is important to investigate whether these proteins are related to cell signals in other cancer processes.

In conclusion, we propose that the co-expression of EZR, CLIC5 and PODXL in liver tissue occurs in pathological conditions, such as HCC, and that these proteins are associated with the increased cell migration and invasive potential of neoplastic cells. CLIC5 may function as a scaffold protein between EZR and PODXL, and the temporal association of this complex with pathological conditions needs to be further characterized.

## Supporting Information

S1 Fig The carcinogen treatment with DEN and 2-AFF in MRHM produced nodules and tumors in rats at 9, 12 and 18 months. A) Schematic representation of Modified Resistant Hepatocyte Model (MRHM). Asterisks show the months that were selected for this study. B-D) Representative control livers at 9, 12 and 18 months respectively. E-F) Representative livers of MRHM at 9, 12 and 18 months respectively. Arrowhead indicates the nodule lesion and the arrows indicate tumors.
(TIF)

S2 Fig EZR, PODXL and CLIC5 expression in nodular regions of MRHM rat livers. Only PODXL consistently co-localized to GSTP-positive lesions. EZR and CLIC5 did not have a consistent expression pattern. (40x magnification).
(TIF)

S3 Fig Evaluation of the specificity of EZR, PODXL and CLIC5 antibodies. Signal in glomeruli and renal tubules of control tissue (kidney) was observed. (20x magnification)
(TIF)

S4 Fig Subcellular localization of EZR, PODXL and CLIC5. The subcellular localization of these proteins was observed in A) HepG2, B) Huh7 and C) SNU387 cells. F-actin is shown in red, and green denotes the proteins of interest. The nuclei are shown in blue. Arrows indicate the location of the proteins of interest in XZ and XY sections.
(TIF)

S1 Table The ARRIVE Guidelines checklist 2014. Animal Research: Reporting In Vivo Experiment.
(PDF)

S2 Table CLIC5, EZR and PODXL immunoreactivity in HCC human biopses. (XLSX)
