# Supplementary figures and images for: Co-Expression of Ezrin-CLIC5-Podocalyxin Is Associated with Migration and Invasiveness in Hepatocellular Carcinoma
Source: PLoS One. 2015 Jul 2;10(7):e0131605. doi: 10.1371/journal.pone.0131605 (PMC4489913; doi:10.1371/journal.pone.0131605)

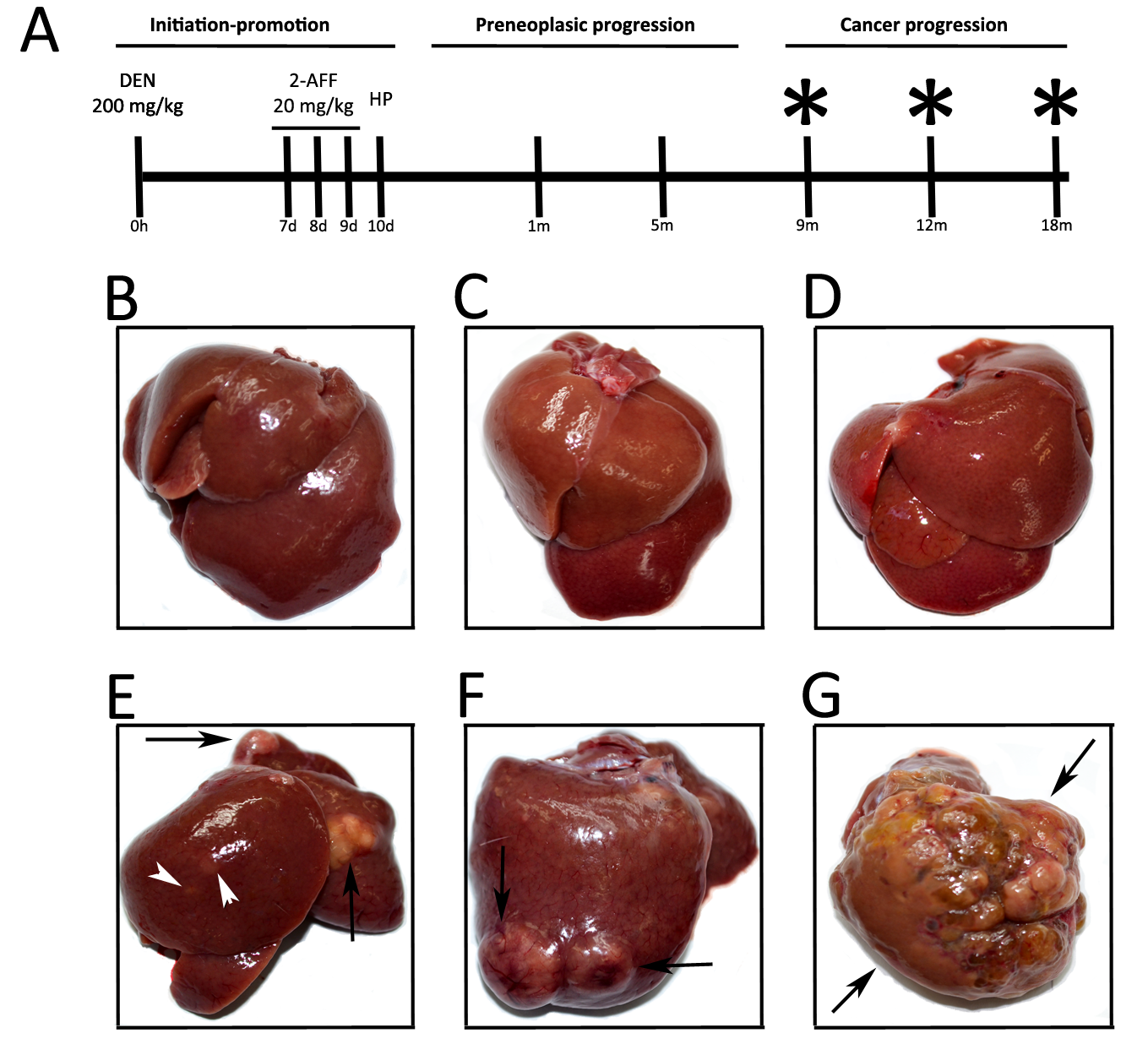

Supplement: S1 Fig — A) Schematic representation of Modified Resistant Hepatocyte Model (MRHM). Asterisks show the months that were selected for this study. B-D) Representative control livers at 9, 12 and 18 months respectively. E-F) Representative livers of MRHM at 9, 12 and 18 months respectively. Arrowhead indicates the nodule lesion and the arrows indicate tumors. (TIF) [file pone.0131605.s001.tif]

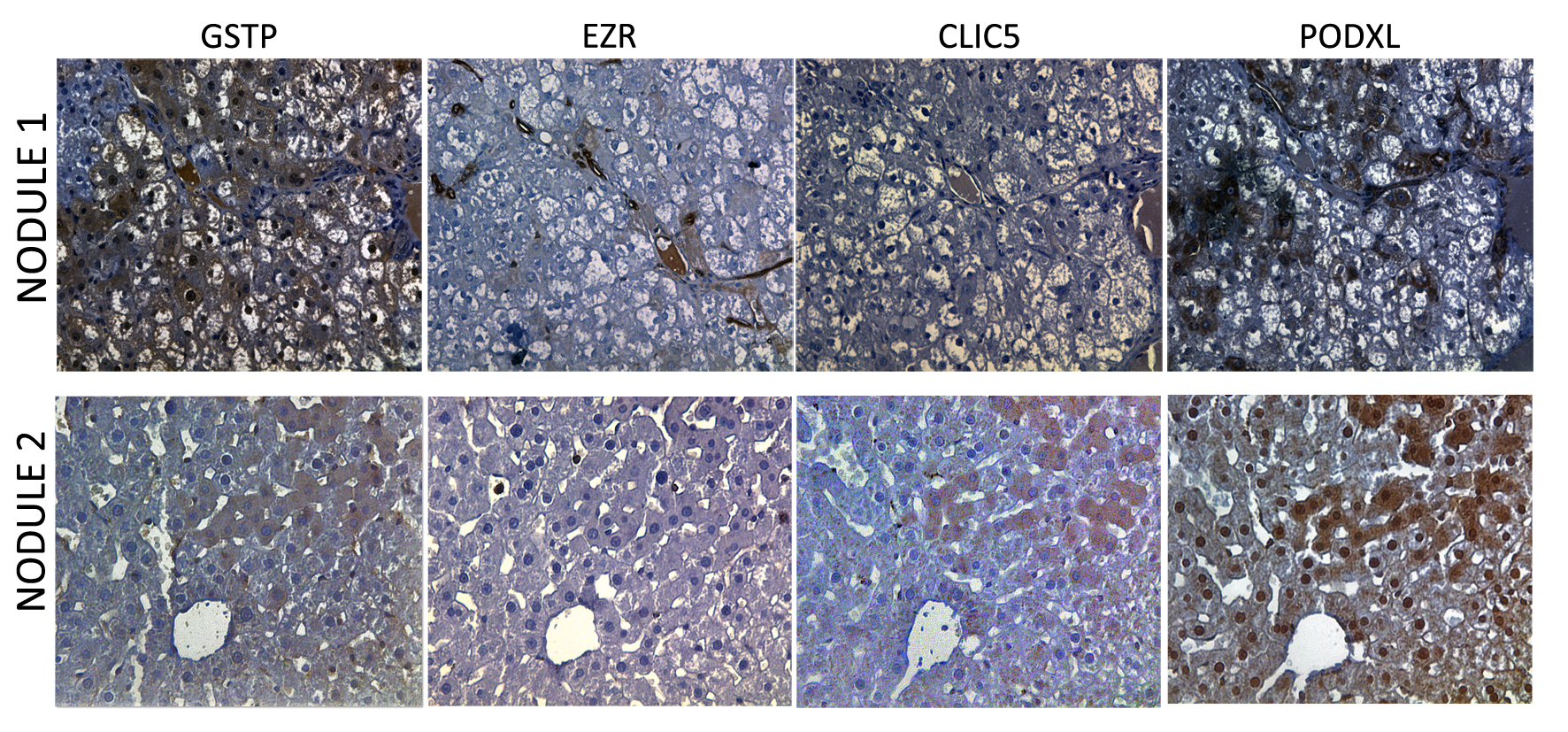

Supplement: S2 Fig — Only PODXL consistently co-localized to GSTP-positive lesions. EZR and CLIC5 did not have a consistent expression pattern. (40x magnification). (TIF) [file pone.0131605.s002.tif]

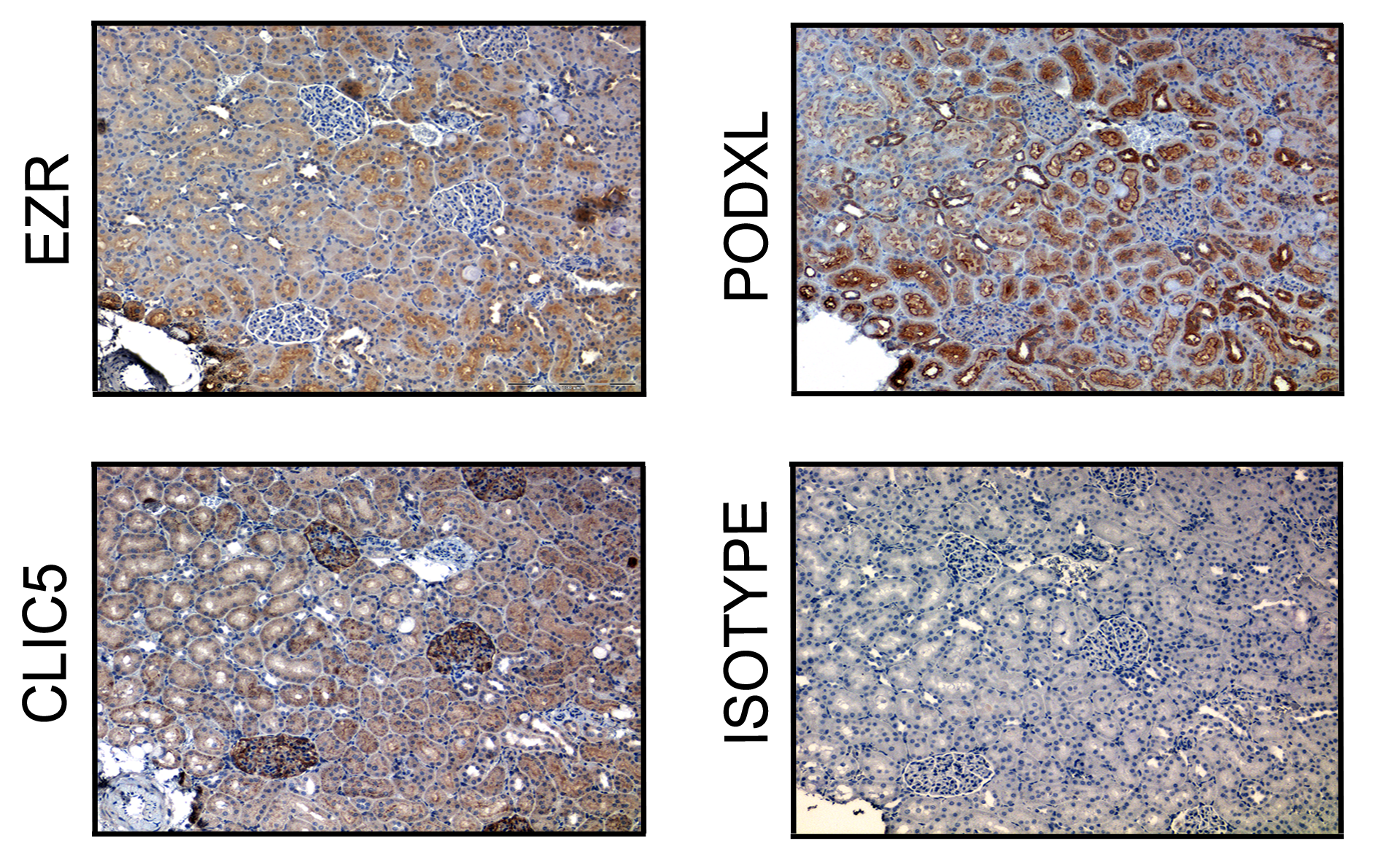

Supplement: S3 Fig — Signal in glomeruli and renal tubules of control tissue (kidney) was observed. (20x magnification) (TIF) [file pone.0131605.s003.tif]

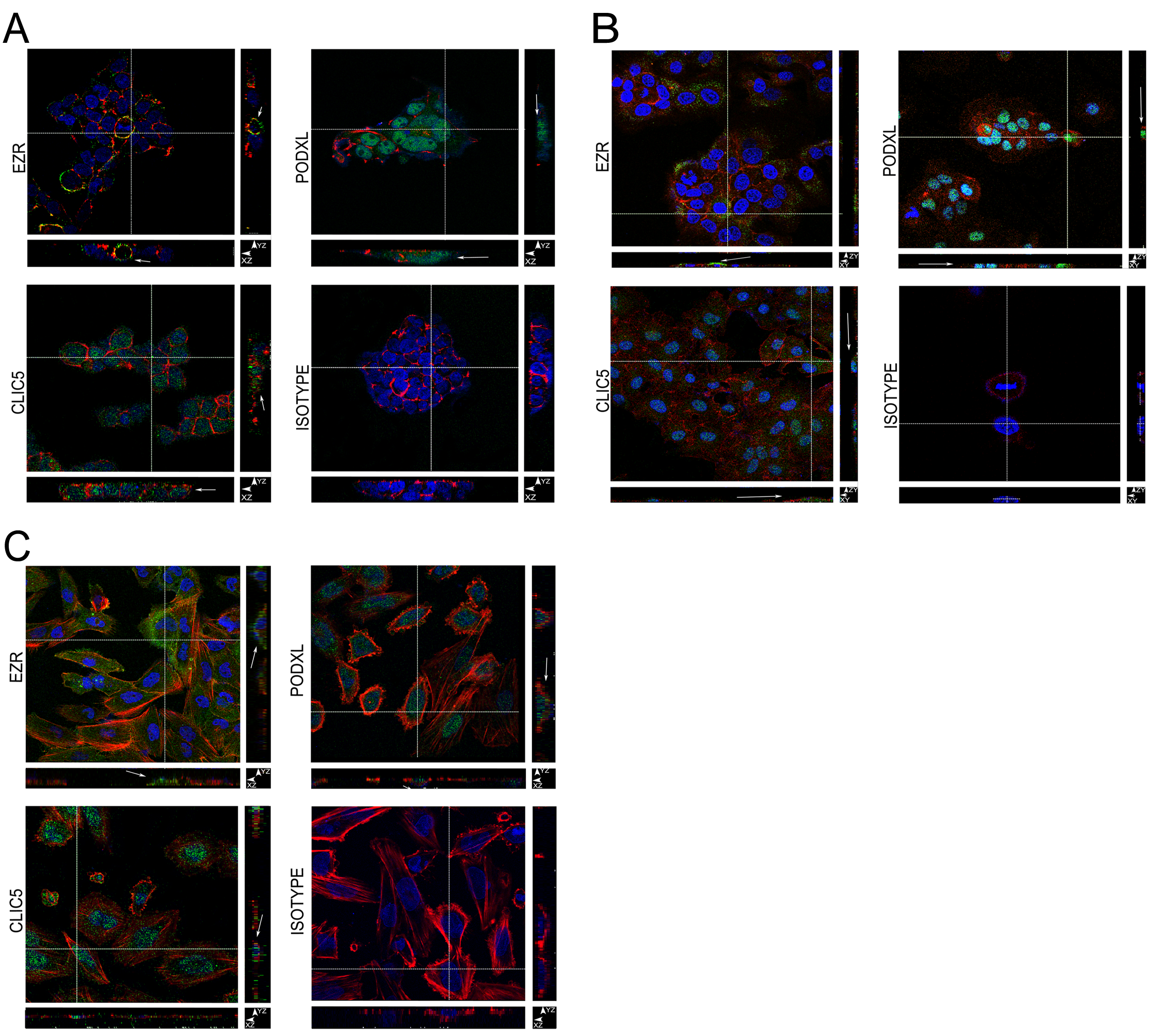

Supplement: S4 Fig — The subcellular localization of these proteins was observed in A) HepG2, B) Huh7 and C) SNU387 cells. F-actin is shown in red, and green denotes the proteins of interest. The nuclei are shown in blue. Arrows indicate the location of the proteins of interest in XZ and XY sections. (TIF) [file pone.0131605.s004.tif]
